# Comparative Analysis of the Vlasiator Simulations and MMS Observations of Multiple X‐Line Reconnection and Flux Transfer Events

**DOI:** 10.1029/2019JA027410

**Published:** 2020-07-22

**Authors:** M. Akhavan‐Tafti, M. Palmroth, J. A. Slavin, M. Battarbee, U. Ganse, M. Grandin, G. Le, D. J. Gershman, J. P. Eastwood, J. E. Stawarz

**Affiliations:** ^1^ Climate and Space Sciences and Engineering University of Michigan Ann Arbor MI USA; ^2^ Laboratoire de Physique des Plasmas (LPP), École Polytechnique, CNRS Sorbonne Université, Institut Polytechnique de Paris Palaiseau France; ^3^ Department of Physics University of Helsinki Helsinki Finland; ^4^ NASA Goddard Space Flight Center Greenbelt MD USA; ^5^ Blackett Laboratory Imperial College London UK

**Keywords:** magnetic reconnection, flux transfer events, Magnetospheric Multiscale Mission, global hybrid‐Vlasov Vlasiator simulations, reconnection‐driven magnetic island dynamics, FTE evolution

## Abstract

The Vlasiator hybrid‐Vlasov code was developed to investigate global magnetospheric dynamics at ion‐kinetic scales. Here we focus on the role of magnetic reconnection in the formation and evolution of magnetic islands at the low‐latitude magnetopause, under southward interplanetary magnetic field conditions. The simulation results indicate that (1) the magnetic reconnection ion kinetics, including the Earthward pointing Larmor electric field on the magnetospheric side of an X‐point and anisotropic ion distributions, are well‐captured by Vlasiator, thus enabling the study of reconnection‐driven magnetic island evolution processes, (2) magnetic islands evolve due to continuous reconnection at adjacent X‐points, “coalescence” which refers to the merging of neighboring islands to create a larger island, “erosion” during which an island loses magnetic flux due to reconnection, and “division” which involves the splitting of an island into smaller islands, and (3) continuous reconnection at adjacent X‐points is the dominant source of magnetic flux and plasma to the outer layers of magnetic islands resulting in cross‐sectional growth rates up to + 0.3 R_E_^2^/min. The simulation results are compared to the Magnetospheric Multiscale (MMS) measurements of a chain of ion‐scale flux transfer events (FTEs) sandwiched between two dominant X‐lines. The MMS measurements similarly reveal (1) anisotropic ion populations and (2) normalized reconnection rate ~0.18, in agreement with theory and the Vlasiator predictions. Based on the simulation results and the MMS measurements, it is estimated that the observed ion‐scale FTEs may grow Earth‐sized within ~10 min, which is comparable to the average transport time for FTEs formed in the subsolar region to the high‐latitude magnetopause. Future simulations shall revisit reconnection‐driven island evolution processes with improved spatial resolutions.

## Key Points

- Anisotropic ion distributions are reported in Vlasiator simulations and MMS observations of reconnection inflow regions
- The 2‐D simulations suggest magnetic islands grow mainly via continuous reconnection. Island coalescence, erosion, and division are also present
- Based on simulation results, ion‐scale FTEs are estimated to grow at < + 0.3 R_E_^2^/min, becoming Earth sized ~10 min after initial formation

## 1 Introduction

Akhavan‐Tafti, Slavin, Eastwood, et al. (2019) classified flux transfer event (FTE) growth mechanisms into two main categories: (1) FTE growth via adiabatic expansion due to decreasing external pressure away from the reconnection region and (2) magnetic reconnection. In the latter category, FTE growth occurs via continuous supply of magnetic flux and plasma to the outer layers of FTEs by reconnection at adjacent X‐lines and/or coalescence with the neighboring FTEs.

**Figure 1:**
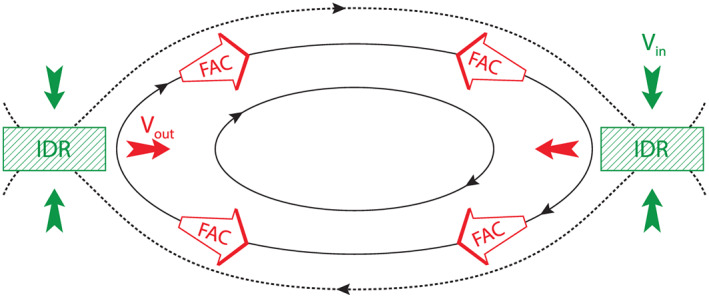
Two dimensional schematics of ion flows in a typical FTE forming due to multiple X‐line reconnection. Field‐aligned currents (FACs; white‐red arrows) are generated due to reconnection. Inflowing ions (*V*_*in*_; green arrow) are also accelerated inside the ion diffusion region (IDR) perpendicular to the magnetic field (*V*_*out*_; red arrow) downstream of an X‐line. The reconnecting field lines, that is, separatrices, are shown as dashed lines.

Figure 1 shows a magnetic island which is a 2‐D projection of a flux rope generated due to primary, multiple X‐line reconnection. The magnetic island can grow via continuous reconnection (Akhavan‐Tafti, Slavin, Eastwood, et al., 2019) at adjacent X‐lines. The X‐lines at the two ends of the magnetic island are represented as ion diffusion regions (IDRs). Inside IDR, inflowing ions (V_in_; green arrows) are demagnetized and accelerated outward as perpendicular jets (solid red arrows) and field‐aligned currents (red‐stroke arrows). The electron diffusion region (EDR), not shown here, is located inside the IDR (Burch & Phan, 2016). Electrons become demagnetized inside the EDR before becoming energized by the reconnection's magnetic‐to‐kinetic energy conversion.

At the subsolar magnetopause, where FTEs are likely generated (e.g., Akhavan‐Tafti et al., 2018; Lee & Fu, 1985), the magnetic field strength and plasma properties of reconnecting field lines are asymmetric across the X‐line. Theory and observations have linked this asymmetry to anisotropic (i.e., non‐Maxwellian) ion and electron velocity distribution functions (VDFs) in the inflow and outflow regions (e.g., Bessho et al., 2016; Burch, James, et al., 2016; Egedal et al., 2011; Hesse et al., 2014).

Sharp spatial gradients, subgyro‐period temporal variations, or sources and sinks in phase space can give rise to nongyrotropic distribution functions. Gyrotropy is a measure of a distribution function's weighted average of variances of velocities perpendicular to the local field direction (Aunai et al., 2013; Che et al., 2018; Swisdak, 2016). Nongyrotropic, also known as agyrotropic, plasma populations depend on gyro‐phase angle, and therefore, they are not in thermal equilibrium. They carry excess energy and may excite unstable waves (e.g., Motschmann et al., 1999). Crescent‐shaped VDFs are a class of nongyrotropic plasma distributions. They are indicative of the reconnection diffusion region and are often observed in spacecraft measurements (Burch, James, et al., 2016; Nagai et al., 2015) and simulations (e.g., Bessho et al., 2016; Hesse et al., 2014).

**Figure 2:**
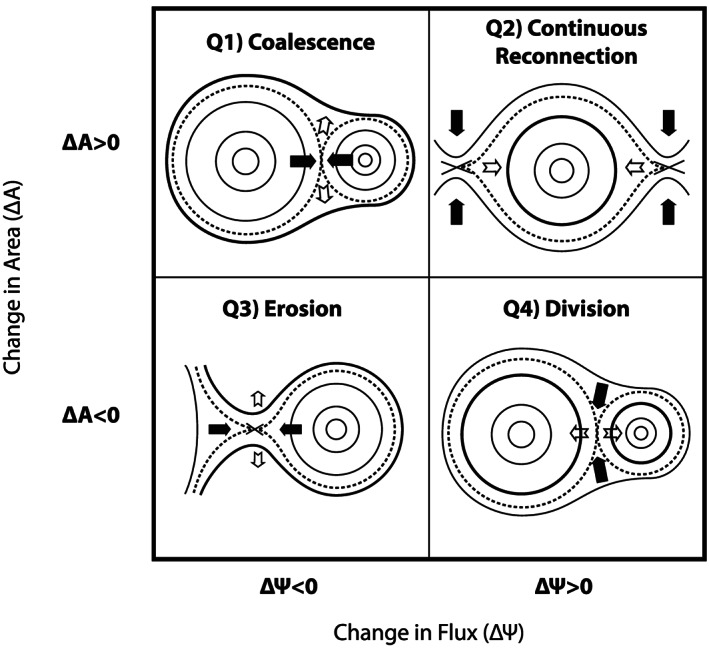
Schematic of magnetic island dynamics driven by magnetic reconnection. The enclosed area, *A*, inside magnetic islands increases due to coalescence and continuous reconnection at adjacent X‐lines. The island's magnetic flux content, *ψ*, changes due to magnetic reconnection. The arrows indicate island convection flows, wherein the inflow is shown in black solid arrows and the outflows are presented as white arrows. Last reconnected field lines are indicated as bold solid lines. The dashed lines represent magnetic separatrices, which embody reconnecting field lines.

Magnetic islands, which are, to a first‐order approximation, two‐dimensional projections of flux ropes, can grow due to magnetic reconnection (e.g., Akhavan‐Tafti, Slavin, Eastwood*,* et al., 2019a). Simulations and observations have also indicated that the cross section of a magnetic island can be reduced due to reconnection (e.g., Hasegawa et al., 2016; Øieroset et al., 2011). The role of reconnection in determining magnetic island dynamics can be divided into four overarching categories: (1) coalescence, (2) continuous reconnection, (3) erosion, and (4) division. Figure 2 represents simplified schematics of the four categories. These reconnection‐driven island dynamics are not necessarily independent and, thus, can take place concurrently. Individual magnetic islands are represented with concentric circles. The last reconnected‐field lines are depicted with bold solid lines, and the newly reconnecting field lines, that is, magnetic separatrix, are shown with dashed lines. The solid and hollow arrows determine the convection speed flowing into and out of the reconnection site, respectively.

In the first category, labeled as Q1, two magnetic islands coalesce. The two neighboring islands merge and create one island whose area, A, is larger than either of the two islands. However, reconnection at the outer layers of the two merging islands will reduce the overall magnetic flux, *ψ*. Therefore, the resulting magnetic island has a larger cross section (A_*product*_ > A_2_, A_1_) and contains equal or greater magnetic flux (*ψ*_*product*_ = *ψ*_*larger island*_ > *ψ*_*smaller island*_ in the two‐dimensional case and *ψ*_*product*_ > *ψ*_*larger island*_ > *ψ*_*smaller island*_ in three‐dimensional coalescing flux ropes with a shear angle) than at least one of the two original islands (cf. Figure 1 in Akhavan‐Tafti, Slavin, Eastwood*,* et al., 2019). The second category, Q2, involves island growth due to continuous reconnection at the adjacent X‐lines. Here the continuous supply of magnetic flux to the outer layers of the magnetic island enlarges the structure, both in terms of magnetic flux and area (*∆ψ* > 0 and *∆*A > 0).

In the other two categories, the overall cross‐sectional area of the magnetic island is reduced over time, due to magnetic reconnection. In these categories, magnetic reconnection either peels off the outer most layers of a magnetic island, called “erosion,” Q3, or it divides a magnetic island into two smaller magnetic islands, known as “division,” Q4 (e.g.,Hasegawa et al., 2016; Øieroset et al., 2011). In both cases, the area of the original magnetic island(s) decreases with time. For instance, during the coalescence process, the smaller island will be eroded by the larger of the two merging islands. Similar processes are also reported in the magnetotail wherein an earthward moving flux rope is eroded when interacting with the geomagnetic field (e.g., Lu et al., 2015; Man et al., 2018; Poh et al., 2019).

Theory and simulation have attempted to determine the dynamics of magnetic islands that are generated by multiple X‐lines reconnection (e.g., Daughton et al., 2009; Dorelli & Bhattacharjee, 2009; Fermo et al., 2010; Uzdensky et al., 2010). In particular, it is essential to understand how these islands develop from birth at small spatial scales to macroscale objects. In practice, simulating long current layers inside which magnetic islands are generated has proven computationally expensive. Global fluid simulations (e.g., Dorelli & Bhattacharjee, 2009; Raeder, 2006) have successfully resolved these large spatial scales. However, until recently (Chen, Tóth, et al., 2017), these simulations were not capable of capturing the small‐scale physics of reconnection and island formation. Global hybrid‐Vlasov simulations of the magnetopause have also demonstrated the small‐scale physics of magnetic island formation and growth (e.g., Hoilijoki et al., 2017, 2019).

In the context of FTE growth, the correlation between the observed normalized reconnection rate and the change in an FTE's magnetic flux content is essential in understanding the rate at which FTEs grow from microscale (Akhavan‐Tafti et al., 2018; Eastwood et al., 2016) to macro‐scale (e.g., Eastwood et al., 2012; Imber et al., 2014; Jasinski et al., 2016; Walker & Russell, 1985) due to reconnection. Normalized reconnection rate is defined as the electric field pointing out of the reconnection plane that drives the reconnection normalized to the reconnecting magnetic field and the local Alfvén speed. However, the normalized reconnection rate from in situ observations cannot determine the rate at which magnetic flux is reconnected globally. The global normalized rate of reconnection is approximately 0.1 (e.g., Cassak et al., 2017) and in situ magnetospheric observations of magnetic reconnection have reported reconnection rates up to 0.2 (e.g., Chen, Hesse, et al., 2017; Fuselier et al., 2005; Genestreti et al., 2018; Mozer et al., 2002; Phan et al., 2007; Slavin et al., 2009), consistent with theory (Cassak & Shay, 2007; Liu et al., 2017).

In the present study, we take advantage of the hybrid‐Vlasov code Vlasiator to study the evolution of magnetic islands at the magnetopause. First, the ion kinetics inside and around two adjacent X‐points are investigated. A first X‐point is selected to demonstrate ion dynamics at a typical magnetopause reconnection. Another X‐point is located in the vicinity and in the downstream of the first X‐point and further sandwiched between two magnetic islands. Next, all magnetic islands, defined as a bundle of magnetic flux function, that is, “O point,” positioned between two saddle points, that is, “X‐points,” are automatically identified with an algorithm. The identified islands are then grouped into four main quadrants based on their temporal change in enclosed magnetic flux and cross‐sectional area, as discussed in Figure 2. The temporal evolution of magnetic islands' enclosed flux and cross‐sectional area is further investigated to estimate the average rate of FTE growth at the magnetopause. Lastly, the Vlasiator simulation results are compared with Magnetospheric Multiscale (MMS) measurements of a series of FTEs embedded in a reconnecting current sheet between two dominant X‐lines at the magnetopause. It is concluded that (i) despite not resolving the ion inertial length in the magnetosheath, Vlasiator simulations capture the ion kinetics in the vicinity of X‐points, thus enabling the study of reconnection‐driven island evolution processes, and (ii) the global magnetospheric Vlasiator simulations indicate that the recently formed, subsolar small‐scale FTEs can grow macroscale at <+0.3 *R*_*E*_^2^/min, while being transported to the high‐latitude magnetopause (e.g., Akhavan‐Tafti et al., 2018).

## 2 Methods

### 2.1 Global Hybrid‐Vlasov Simulation Code Vlasiator

The hybrid‐Vlasov code Vlasiator (http://www.helsinki.fi/en/researchgroups/vlasiator) has been developed to investigate global magnetospheric dynamics at ion‐kinetic scales (Palmroth et al., 2018; von Alfthan et al., 2014). Vlasiator solves the Vlasov equation, evolving ions (protons) as distribution functions in three velocity‐space dimensions, tracked on a cartesian grid. Electrons are treated as a cold massless charge‐neutralizing fluid, and closure is provided by the generalized Ohm's law including the Hall term. The simulation used in this study is two dimensional in real space and three dimensional in velocity space (Grandin et al., 2019; Hoilijoki et al., 2017; Jarvinen et al., 2018; Juusola, Hoilijoki, et al., 2018; Juusola, Pfau‐Kempf, et al., 2018; Palmroth et al., 2013, 2017).

The simulation domain extends within −94 *R*_*E*_ < *X* < +48 *R*_*E*_ and −56 *R*_*E*_ < *Z* < +56 *R*_*E*_, where *R*_*E*_ = 6,371 km is the Earth's radius. The geocentric solar ecliptic (GSE) coordinates are used in which X‐points sunward, Y points opposite Earth's motion about the Sun, and Z points normal to the ecliptic plane. The inner boundary with a radius of 5 *R*_*E*_ is modeled as an ideal conducting sphere. Due to the two‐dimensional nature of the simulation in the ordinary space, the dipole field is implemented as a 2‐D line dipole with a strength resulting in a realistic magnetopause standoff distance (Daldorff et al., 2014). The simulation initialization process involves distributing plasma with a stationary Maxwellian distribution within the simulation domain's inner boundary. The simulation is carried out for 2,150 s of simulation time. The analysis provided in this study only includes the final 1,050 s of the simulation run.

We model a steady solar wind inflow at the +*x* boundary, with a fast solar wind of **v** [km/s] = −750 $\hat{x}$, a density of *n*_*sw*_ = 1 cm^−3^, a proton temperature of *T*_*p*_ = 0.5 MK, and a purely southward interplanetary magnetic field (IMF) of magnitude 5 nT. The fast solar wind is intended to speed up the initialization of the simulation run. The 2‐D spatial grid resolution is 300 km in each direction, and the 3‐D velocity space grid is 30 km/s in each direction.

The magnetosheath ion inertial length (*d*_*i*_ ~ 150 km at the magnetopause under the stated upstream conditions) is not resolved in the Vlasiator's simulation grid (spatial grid resolution = 300 km). However, in previous Vlasiator studies, such as in the modeling of collisionless shock kinetics by Pfau‐Kempf et al. (2018), it was confirmed that even when drastically underresolving ion inertial scales (spatial grid resolution >8 *d*_*i*_), Vlasiator simulations can successfully capture ion kinetic effects. In this study, we further investigate ion kinetics in magnetopause reconnection in two‐dimensional Vlasiator simulations. Previous global simulations, such as magnetohydrodynamic (MHD) with embedded particle‐in‐cell (PIC) simulations (Chen, Tóth, et al., 2017), have reproduced some kinetic features associated with magnetopause reconnection.

The Vlasiator simulations use physical scale lengths to model the Earth's magnetosphere, unlike other global simulations, where characteristic scale lengths are scaled up (e.g., Tóth et al., 2017). In addition, Vlasiator simulations provide noise‐free VDFs, which are essential in propagating physical particle distributions in anisotropic plasma regimes, such as the magnetosheath (e.g., Gary et al., 1993). A global kinetic approach such as that of Vlasiator further avoids contamination of dynamics from MHD‐kinetic boundary effects.

### 2.2 Observational Instrumentation

The four identical MMS spacecraft were launched in 2015 and are designed to unravel the physics of magnetic reconnection (Burch & Phan, 2016). The high temporal and spatial plasma measurements (Fast Plasma Investigation (FPI); Pollock et al., 2016) provide three‐dimensional electron and ion distributions. Plasma moments are constructed from all‐sky FPI electron and ion distributions at 30 and 150 ms cadence, respectively. The fields instrument suites (Torbert et al., 2016) including the fluxgate magnetometers (Russell et al., 2016) and the electric dual probes (Ergun et al., 2016; Lindqvist et al., 2016; Torbert et al., 2016) are used for magnetic and electric field measurements. Multipoint analysis techniques (Harvey, 1998) are used to determine spatial gradients in fields and plasma measurements. The spacecraft separation was, on average, 10 km for our case study (Phase 1a; Burch, Moore, et al., 2016).

## 3 Results

### 3.1 Hybrid‐Vlasov Simulation Results

#### 3.1.1 Vlasiator Case Study

As Vlasiator simulations operate directly on a mesh‐based representation of 6‐D phase space, velocity space plots are taken straight from the instantaneous simulation state. The VDF plots are constructed within a single simulation grid cell at each individual timestep. The VDF plots presented here show rebinned slices of velocity space in the local magnetic frames, with the local plasma bulk velocity subtracted, identical to the MMS spacecraft measurements.

Due to the sparse velocity space representation in Vlasiator (von Alfthan et al., 2014), cells with a phase space density of less than 10^−15^ s^3^ m^−6^ are dropped from the simulation, except when neighboring denser regions are present. Dropped regions are displayed as empty areas in the VDF plots.

**Figure 3:**
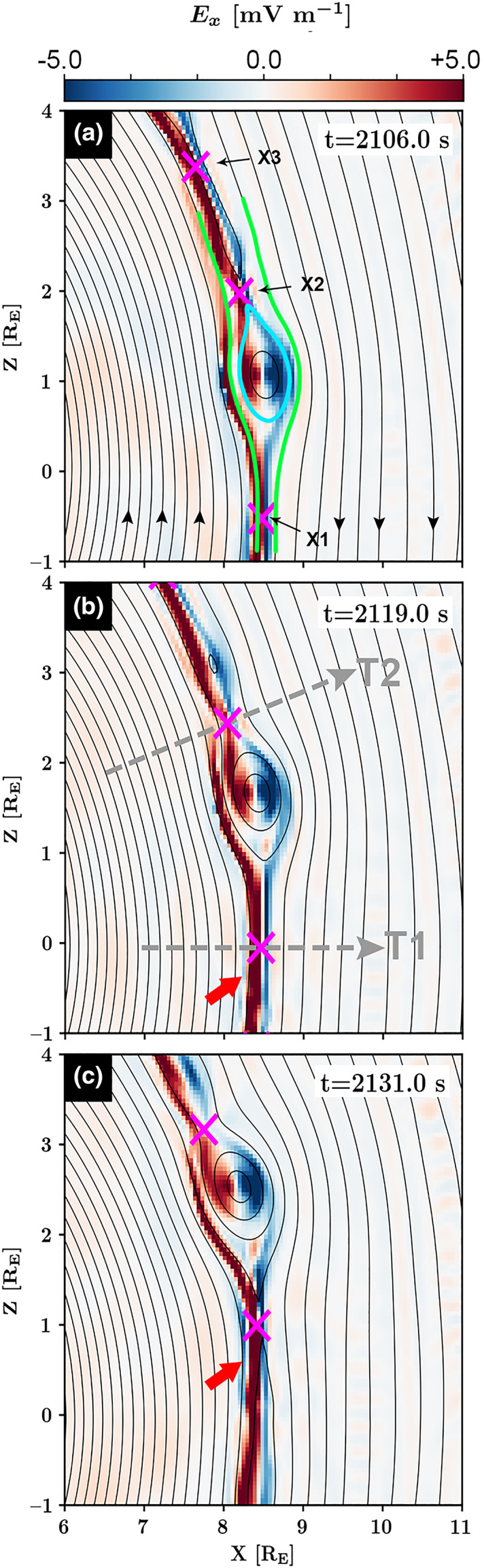
Three simulation timeframes (simulation box: 6 < *X* [RE] < 11 and −1 < *Z* [RE] < 4 at *t* = 2,106.0, 2,119.0, and 2,131.0 s since the simulation initialization) to illustrate the temporal evolution of a magnetic island in the northern hemisphere magnetosheath. The color bar indicates *E*_*x*_. The magenta “X” markers indicate the X‐points, that is, saddle points in the magnetic flux function. The reconnecting field lines are shown in green. The newly formed magnetic island located between X1 and X2 is shown in cyan. The dashed lines in panel b provide two virtual spacecraft trajectories, T1 and T2.

Figure 3 provides three simulation timeframes to illustrate the formation and temporal evolution of a magnetic island, defined as a bundle of magnetic flux function, that is, “O point,” positioned between two saddle points, that is, “X‐points” shown in magenta, along the Northern Hemisphere magnetopause. Initially (not shown here), the last open magnetosheath field line reconnects with the last closed magnetospheric field line at X1. Shortly after at *t* = 2,106.0 s, as shown in panel a, the newly opened field line reconnects at X2, resulting in the generation of a magnetic island (diameter ~1 *R*_*E*_), shown in cyan. The reconnecting field lines between the two simultaneous X‐points, X2 and X3, further generates an island between them, as shown by a small O point sandwiched between X2 and X3 in panel b. Field lines shown in green continue to reconnect at X1 and X2, causing the cyan magnetic island to grow with time, panels a–c, while convecting northward along the magnetopause.

Magnetic islands are shown to form between two X‐points. The onsets of the two neighboring X‐points may occur at the same time (small island in Figure 3b) or at different times (cyan island in Figure 3a). The latter was originally proposed by Raeder (2006) and named as the sequential multiple X‐line reconnection (SMXR) model. In the case of islands generated by SMXR, the islands are embedded in field lines that are on one side connected to the magnetosphere and on the other to the magnetosheath and the solar wind. Also, as shown in Figure 3a, both the (cyan) island and X2 are located in the exhaust region of X1. The spatial and temporal variations between X1 and X2 indicate that magnetic flux must first reconnect at X1 before rereconnecting at X2. Therefore, X2 reconnects only the magnetic flux that has already been reconnected at X1.

**Figure 4:**
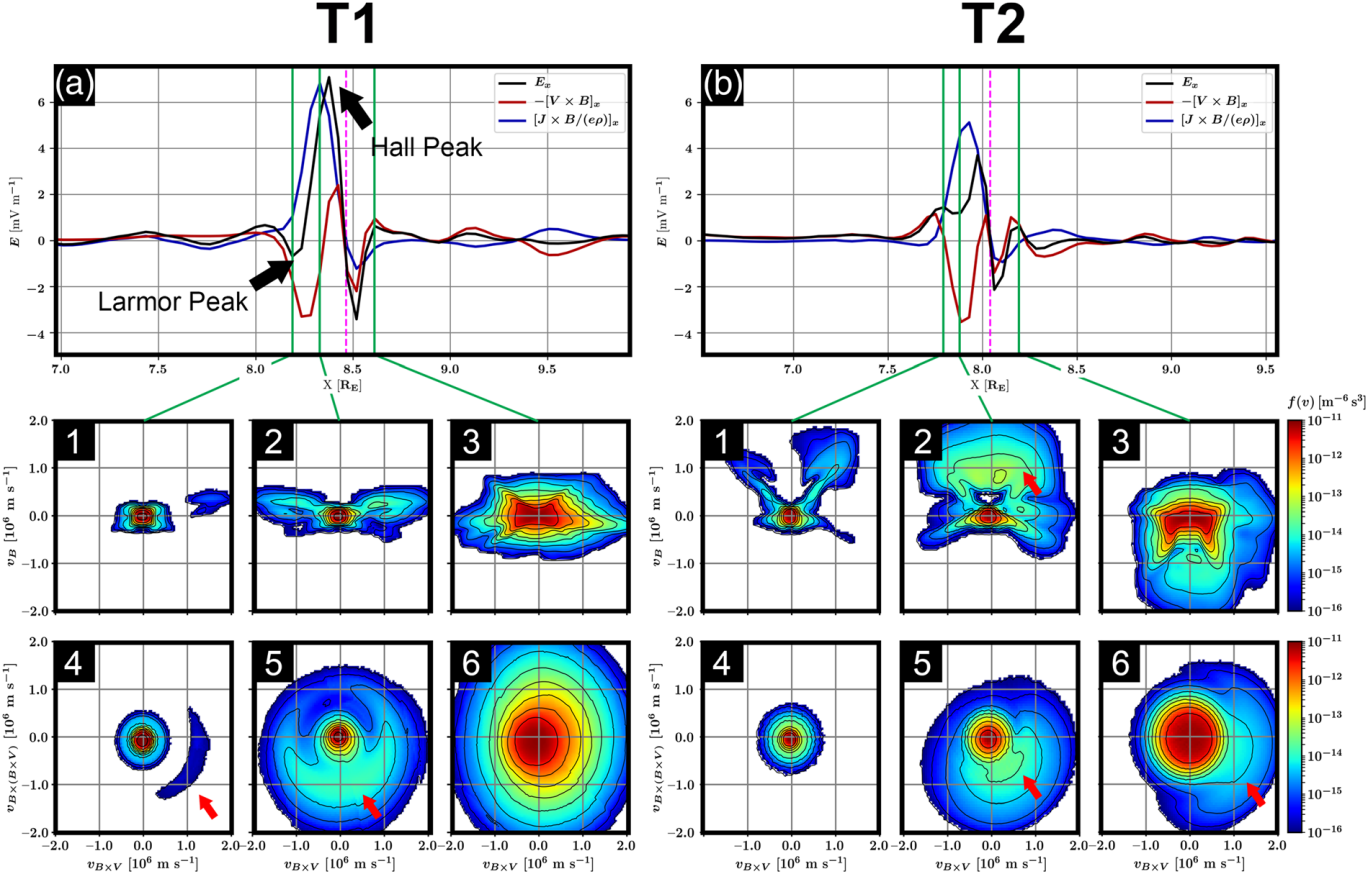
Vlasiator electric field profiles and ion velocity distribution functions across X1 and X2 along two virtual spacecraft trajectories, T1 and T2. The panels include (a and b) *E*_*x*_ profile (black solid curve) along virtual spacecraft T1, including the Ohm's law components, in particular, the convection term (−V × B; red solid curve) and the hall term (J ×B/ne; blue solid curve), and (1–3) ion VDFs sliced in the **V**_B_ − **V**_B × V_ and (4–6) **V**_B × V_ − **V**_B × (B × V)_ planes, where **V**_B_ represents the velocity along the magnetic field orientation, **V**_B × V_ and **V**_B × (B × V)_, respectively, along (**B** × **V**) and **B** × (**B** × **V**) directions, where **V** is the ion bulk velocity. The ion VDFs are generated at different *E*_*x*_ extrema and marked with vertical green solid lines. The X‐point crossing is marked with a vertical magenta dashed line. Two videos illustrating the temporal evolutions of ion VDFs as a function of *E*_*x*_ and position with respect to X1 and X2 are included in the supporting information Videos S1 and S2, respectively.

The ion VDFs and electric field signatures in the vicinity of X1 and X2 are examined along two virtual spacecraft trajectories, labeled as “T1” and “T2” in Figure 4. The panels include (a and b) *E*_*x*_ profile (black solid curve) along virtual spacecraft T1 and T2, including the Ohm's law components, in particular, the convection term (−v ×B; red solid curve) and the Hall term (J×B/ne; blue solid curve), and (1–3) ion VDFs sliced in the **V**_B_ − **V**_B × V_ and (4–6) **V**_B × V_ − **V**_B × (B × V)_ planes, where **V**_B_ represents the velocity along the magnetic field orientation, **V**_B × V_ and **V**_B × (B × V)_, respectively, along (**B** × **V**) and **B** × (**B** × **V**) directions, where **V** is the ion bulk velocity. Each ion VDF is generated at a specific *E*_*x*_ extrema, marked in panel a or b with a vertical green solid line. The X‐point crossing is marked with a vertical magenta dashed line.

Trajectory T1 demonstrates *E*_*x*_ signatures that are associated with a nominal magnetosheath X‐point. In particular, there exists a local minimum along *E*_*x*_ on the magnetospheric side of the X‐point, signified by a black arrow as the “Larmor Peak.” Malakit et al. (2013) proposed the formation of a Larmor electric field, defined as an in‐plane electric field in collisionless asymmetric magnetic reconnection, associated with finite Larmor radius effects. Larmor electric field is independent of the Hall electric field. As theory suggests, the Larmor electric field is situated within, the narrow blue shaded regions indicated by red arrows in Figures 3b and 3c, located in the inflow region of the dissipation region where electromagnetic energy conversion occurs (Pritchett & Mozer, 2009). Under close inspection, weak Earth‐directed electric fields can be found intermittently along the last closed field lines, but the strongest signal is found close to X1. The ion Larmor radius in this region is on the order of ~100 km, which again shows how Vlasiator captures kinetic physics, despite not resolving the relevant spatial scales. The ion Larmor radius increases at the magnetopause and at the X‐lines (>300 km).

Larmor electric field is proposed to exist on the magnetospheric side of a collisionless asymmetric magnetic reconnection pointing toward Earth away from the X‐line, that is, *E*_*x*_ < 0. The Vlasiator simulation results indicate the presence of an Earthward electric field extremum, Larmor peak *E*_*x*_ ~ 0.5 mV/m located upstream and on the magnetospheric side of X1. This value is comparable to the theoretical magnitude of the Larmor electric field is estimated to be: *E*_*x*_ ~ k_B_ *T*_*i*_/*q*_*i*_ *r*_*i*_ = 2 mV/m (Malakit et al., 2013), where *k*_*B*_, *T*_*i*_, *q*_*i*_, and *r*_*i*_, respectively, represent the Boltzmann constant and ion magnetospheric temperature, electric charge, and gyro‐radius.

On the magnetospheric side of X1, the Larmor peak is followed by a strong sun‐ward Hall electric field peak, labeled in Figure 4a as the “Hall Peak.” On the magnetosheath side (*X* > 8.4 *R*_*E*_), the x components of the Hall and the convection terms are unidirectional, resulting in an Earthward electric field followed by a positive peak.

The **V**_B_ − **V**_B × V_ VDF slice at the Larmor Peak in Figure 4 (panel a1) shows an intense and uniform core ion population, **V**_B_ < 250 km/s. Panel a4 further indicates the formation of an nongyrotropic ion population in the **V**_B × V_ and **V**_B × (B × V)_ VDF slice. The identified nongyrotropic ion population, indicated by a red arrow in panel a4, corresponds to the so‐called perpendicular crescent‐shaped ion distribution, reported in association with the local *E*_*x*_ minimum, that is, Larmor electric field (e.g., Lapenta et al., 2017). Closer to the X‐point, where (*E*_Hall_)_*x*_ reaches a maximum, the perpendicular crescent‐shaped distributions of the higher‐energy ions, **V**_B × V_ > 250 km/s, becomes clearer, as indicated by a red arrow in panel a5. On the magnetosheath side of X1, where *E*_*x*_ reaches a positive maximum, the ion VDF becomes magnetosheath like and nearly isotropic.

At X2, the electric field profile is more complex. X2 is located in the exhaust region of X1. Compared to X1, X2 is located between two magnetic islands. Figure 4b provides the *E*_*x*_ profile along trajectory T2. The Larmor electric field, that is, the earthward *E*_*x*_, is absent on the magnetospheric side of X2. Instead, *E*_*x*_ has two peaks on the magnetospheric side of X2, resulting from a strong Hall electric field component. The outer *E*_*x*_ peak at X_GSM_ ~ 7.78 *R*_*E*_, is a result of the enhanced ion convection (−V × B; red solid curve) along the X1 separatrix, while the peak at *X*_GSM_ ~ 7.95 *R*_*E*_, is supported by the Hall electric field at X2.

The ion **V**_B_ − **V**_B × V_ VDF slices are different between trajectories T1 and T2. The nongyrotropic ion population in panel a4 is absent at the outer *E*_*x*_ peak, in panel b4. Parallel D‐shaped ion distribution is found in panel b2 and marked by a red arrow. D‐shaped ion VDFs are often observed in the reconnection exhaust region (e.g., Cowley, 1995; Phan et al., 2004). The parallel D‐shaped ion distribution indicates that X2 is located within and is influenced by the outflow exhaust of X1. Perpendicular crescent‐shaped ion distributions also appear closer to the X‐point, as indicated by a red arrow in panel b5, similar to panels a5. When crossing X2, the VDFs transition from magnetospheric type into magnetosheath type as suggested by the high abundance of energetic ions, **V**_B × V_ > 500 km/s. However, unlike the magnetosheath VDFs along trajectory T1, panels b3 and b6 show strongly anisotropic ion distributions. In particular, a nongyrotropic ion population is identified and marked by a red arrow in panel b6, coinciding with an enhanced antiparallel population of ions in panel b3.

**Figure 5:**
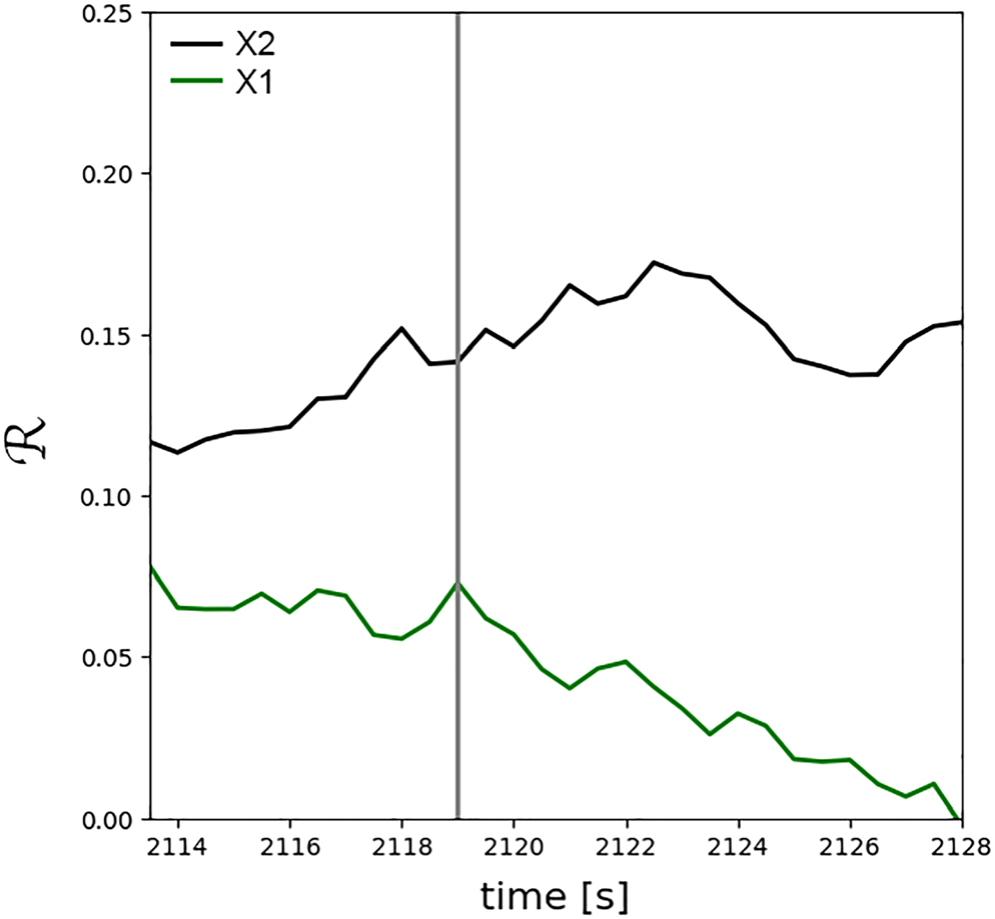
Temporal profile of the normalized reconnection rate, $ℛ=E_{y}^{x‐\text{point}}\;v_{\mathrm{Ai}}^{-1}\;B^{-1}$, at the X1 and X2, in the reference frame of the X‐points. The vertical gray line indicates the simulation timeframe *t* = 2,119.0 s.

The presence of magnetic islands near X2 may impact the rate at which field lines enter the diffusion region. This can be done via (1) building‐up pressure in the reconnection exhaust, that is, inside islands, and/or (2) thickening the current sheet layer within which the islands propagate. The latter can result in increasing magnetic field tension along the highly bent reconnecting field lines, therefore, introducing stress against the inflow flux. Figure 5 shows the rates of reconnection at X1 and X2 as a function of simulation time. The same two X‐points, that is, saddle points in the magnetic flux function, are tracked between simulation frames and their reconnection rates are determined at each time frame, similar to Hoilijoki et al. (2017). The normalized reconnection rate here is defined as the out‐of‐plane electric field component, *E*_*y*_, at the X‐point, normalized by the inflow plasma Alfvén speed, *v*_*Ai*_, and magnetic field magnitude, B, given thus as

$$ℛ=E_{y}^{x‐\text{point}}\;v_{\mathrm{Ai}}^{-1}\;B^{-1}$$

where the inflow values are gathered from the simulation just upstream of the X‐point. The out‐of‐plane component of the electric field, *E*_*y*_, are offset ( $E_{y}^{x‐\text{point}}=E_{y}+V_{X‐\text{point}}\times B$) by the convection term, *E* =  − V_*X‐point*_ × B, where V_*X‐point*_ refers to the X‐point's convection speed at the magnetopause. V_*X‐point*_ is calculated by assuming the instantaneous plasma bulk velocity to describe the motion of the X‐point in space. In order to smooth out the normalization impact of magnetosheath fluctuations such as mirror modes, visible in B and *v*_*Ai*_ signatures (not shown here), the inflow values are averaged over five consecutive simulation cells in the radial direction, at least 1 *R*_*E*_ outward from each X‐point. Consistent with the latest kinetic predictions (e.g., Liu et al., 2017), it is discovered that normalized reconnection rate can surpass the MHD threshold normalized reconnection rate of 0.1 (Cassak et al., 2017). It is further revealed that the normalized reconnection rate at X2, which is located in the exhaust region of X1 and sandwiched between two islands, is larger than X1. In addition, the normalized reconnection rate at X2 increases with time, while the normalized reconnection rate slowly decreases at X1.

#### 3.1.2 Statistical Analysis

In the previous section, it was shown how the Vlasiator code, despite not fully resolving the ion inertial length in the magnetosheath, successfully captures key ion kinetics associated with magnetic reconnection, such as anisotropic ion distributions in the inflow and outflow regions. Thus, the Vlasiator code provides, for the first time, the opportunity to study reconnection‐driven magnetic island evolution processes globally at the magnetopause. In this section, magnetic islands near the subsolar magnetopause are identified using an automated algorithm and their temporal evolution due to reconnection are examined.

**Figure 6:**
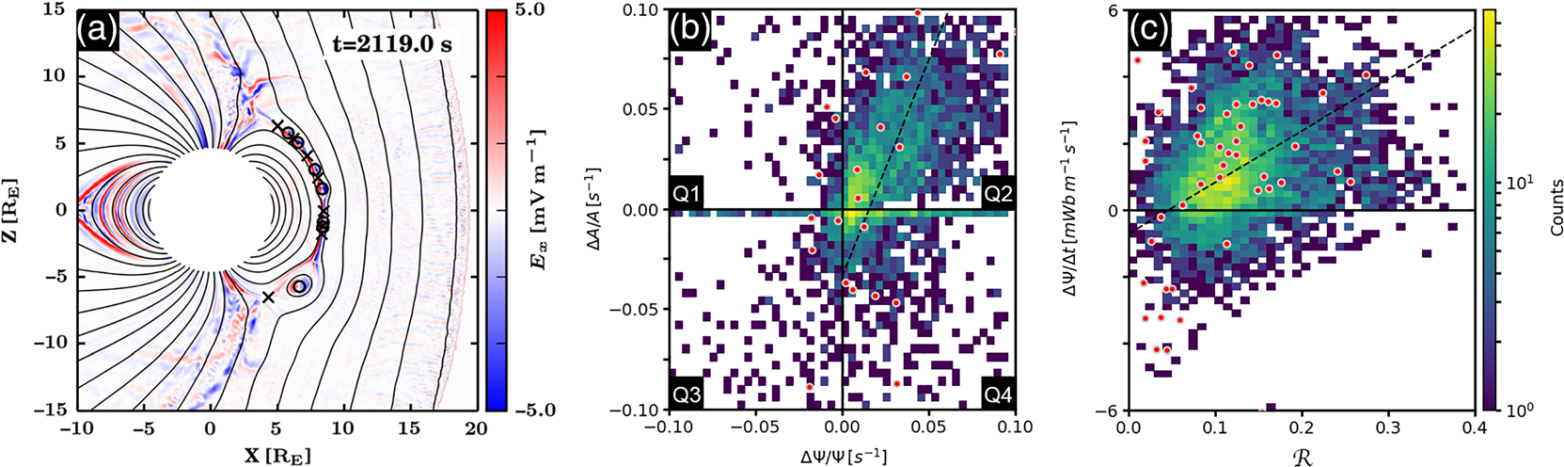
(a) Example snapshot (simulation timeframe: *t* = 2,119.0 s) of the Vlasiator magnetopause. The “X” and “O” points are automatically identified by the algorithm. The color bar indicates the electric field along the X_GSM_ axis, *E*_*x*_ [mV/m]. (b) Magnetic islands categorized into four quadrants, Q1–4, based on their temporal change in enclosed magnetic flux, ∆ψ, and cross‐sectional area, ∆A. The linear fit from the orthogonal distance regression, shown as dashed black line, is *y* = 2.16 × −0.03. The color bars indicate the count of events per bin. (c) The change in the enclosed magnetic flux as a function of normalized reconnection rate, $ℛ=E_{y}^{x‐\text{point}}\;v_{Ai}^{-1}\;B^{-1}$. The linear fit from the orthogonal distance regression, shown as dashed black line, is *y* = 8.6 × −0.5. The red circles denote coalescing magnetic islands wherein two neighboring magnetic islands merge and create one larger island.

Magnetic islands within the simulation are identified by searching for maxima and saddle points of the magnetic flux function (Hoilijoki et al., 2017; Yeates & Hornig, 2011). A magnetic island is defined as encapsulated loops of magnetic field lines, identified as a local maximum of the magnetic flux function (an O point) positioned between two saddle points (X‐points). In other words, we require magnetic islands to be bounded by at least two X‐points (Hoilijoki et al., 2019). Figure 6a illustrates an example simulation timeframe of the dayside magnetosphere in which the “X” and “O” points are automatically identified with an algorithm. The algorithm is also capable of determining the magnetic flux, *ψ*, and the cross‐sectional area, A, for the magnetic islands. The algorithm can further track the merging of islands, the process known as coalescence, and can distinguish X‐points within the islands. We focus on subsolar magnetic islands, tracking only those where the O point is within polar angle, |*θ*| < 30° from the X_GSE_ axis.

Magnetic islands are categorized based on the relative significance of the X and O points. For each island, that is, O point, the two dominant X‐points are defined as the two saddle points with lowest flux function value, that is, which have reconnected the most. O points of an island, defined as a local maximum in the magnetic flux function, are located between two dominant X‐points. The dominant O point is defined as the O point with the highest flux function value. There may exist additional X‐points between two dominant X‐points indicating the presence of interior islands within the dominant structure. Similar structures have been postulated (e.g., Fermo et al., 2011) and were recently observed by MMS (e.g., Hwang et al., 2018). Additionally, the relative location of dominant O and X‐points are tracked between simulation frames. This enabled the identification of island coalescence events in which two neighboring O points merge to create one larger island. During this process, the innermost of three dominant X‐points describing two islands becomes a nondominant inner X‐point. The coalescence process is of great practical significance; hence, it has been the subject of numerous theoretical (e.g., Pritchett & Wu, 1979), experimental (e.g., Yamada et al., 1990), and observational (e.g., Wang, Lu, et al., 2016; Zhao et al., 2016; Zhou et al., 2017) studies.

Table 1 summarizes the distribution of the two‐dimensional magnetic islands identified at the magnetopause. A total of 4,786 magnetic islands, defined as an O point positioned between two X‐points, are independently identified in one simulation run at different locations (|*θ*| < 30°) and different timeframes. This approach is different from Hoilijoki et al. (2019), wherein individual magnetic islands were tracked across all timeframes. The O points are divided by the algorithm into four main categories depending on their structure and evolution: (1) “two X‐points” wherein reconnection at two dominant X‐points forms a magnetic island, (2) “>2 X‐points” in which reconnection at two dominant X‐points forms an O point inside which multiple O points and one or more additional X‐points exist, (3) “Coalescence,” which describes the merging of two independent magnetic islands during which three dominant X‐points (associated with two category 1 islands), are reduced to two dominant X‐points, and (4) “Division,” which describes the process through which one magnetic island is divided into two independent magnetic islands, therefore, a new dominant X‐point is formed between two existing dominant X‐points. The categories 3 and 4 are subcategories of the categories 2 and 1, respectively.

**Table 1:**
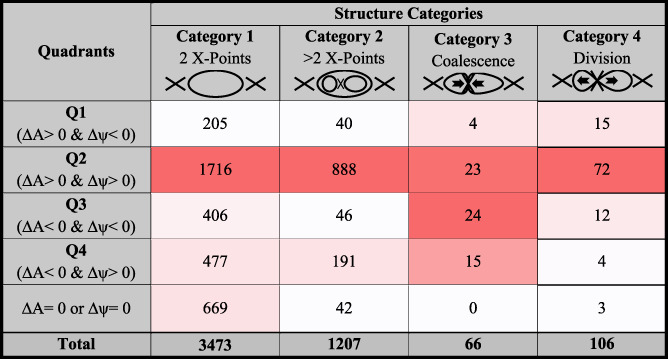
The Number of Islands Included in This Study Partitioned Into Four Main Categories Depending on Their Structure and Evolution

*Note*. The four categories include (1) “2 X‐points” wherein reconnection at two dominant X‐points forms a magnetic island, (2) “>2 X‐points” in which reconnection at two dominant X‐points forms a magnetic island inside which multiple smaller islands and X‐points exist, (3) “Coalescence,” which describes the merging of two independent magnetic islands during which three dominant X‐points are reduced to two dominant X‐points, and (4) “Division,” which describes the process through which one magnetic island is divided into two independent magnetic islands, therefore, two dominant X‐points become three dominant X‐points. The structures are further divided into based on their evolution, describing the change in individual magnetic island's magnetic flux, *∆ψ*, and area, *∆A*. The shade of red indicates the relative magnitude of each cell compared to the column's total counts (bottom row), with bright red signifying the largest value.

In this part of the study, we focus on the evolution of magnetic islands. In particular, we are interested in reconnection‐associated processes that contribute to a change in a magnetic island's dimensions and/or flux content between two consecutive timeframes. This is achieved by identifying all magnetic islands in a given simulation timeframe, independent of other timeframes, and tracking the identified magnetic islands in a subsequent timeframe to study the change in dimensions and/or flux content between timeframes. In practice, the evolution of an island through its lifetime can involve a multitude of the above mechanisms. However, for the purposes of this study, only the evolution processes between two consecutive timeframes are considered.

The magnetic islands are split into four main quadrants, Q1–4, based on the temporal change in magnetic flux content, *∆ψ*, and cross‐sectional area, ∆A, as described in Figure 2. To achieve this, the flux content and the cross‐sectional area of magnetic islands in each simulation frame are compared with the previous frame (or subsequent frame; in the case of island division). There remains a subpopulation of islands whose cross‐sectional area does not change between two consecutive time frames. The islands in which the magnetic flux content and/or the cross‐sectional area does not change between simulation frames are separated from the four quadrants and listed in the table as (*∆A* = 0 and/or *∆ψ* = 0). Our observations include

1. Continuous reconnection at adjacent dominant X‐points supplies additional magnetic flux, *∆ψ* > 0, to the outer layers of the majority of “uniform” magnetic islands, defined as islands with no substructure, (Category 1, Q2 and Q4; 2193/3473 ~ 63%), and those with substructure (Category 2, Q2 and Q4; 1079/1207 ~ 89%). The sizes of these islands also increase, (*∆A* > 0; Q1 and Q2), in nearly 55% and 77% of islands in Categories 1 and 2, respectively.
2. Nearly 16% of islands with substructure (Category 2) exhibit simultaneous increase of magnetic flux content and reduction of cross‐sectional area, *∆A* < 0 (Q4).
3. The coalescence process, Category 3, is more complex. In 41% of the cases (Q1 and Q2), it involves the growth of the total enclosed area (*∆A* > 0, *A*_*coalescence*_ > *A*_*pre,* 1_ and *A*_*pre,* 2_, where *A*_*coalescence*_, *A*_*pre,* 1_, and *A*_*pre,* 2_ represent cross‐sectional areas of islands after and before the coalescence process, respectively). Similarly, 36% of the islands are found to “erode” (*∆A* < 0 and *∆ψ* < 0; Q3) when coalescing, Category 3, with larger islands. Moreover, 23% of the islands are found to compress (*∆A* < 0 and ∆*ψ* > 0; Q4) during the coalescence process.
4. Magnetic island division, Category 4, involves the splitting of one magnetic island into two smaller islands (and in one case, three islands). The likelihood of identifying island division events is nearly twice that of island coalescence events suggesting that the division of magnetic islands is more common than coalescence (e.g., Øieroset et al., 2011, 2019).
5. Magnetic flux and area of dividing islands, Category 4, are found to increase (*∆A* > 0 and *∆ψ* > 0; Q2) in the majority of cases, in contrary to the proposed classification in Figure 2. Similarly, magnetic flux is found to increase in more than 50% of the coalescing islands. Island area decreases in nearly 60% of the coalescing islands. As discussed above, when coalescing, the minor island erodes (*∆A* < 0 and *∆ψ* < 0), contributing to the growth, in both area and magnetic flux content, of the larger island.
6. Most importantly, >70% of the islands evolve due to continuous reconnection only, Category 1, further emphasizing the significance of magnetopause reconnection with the interplanetary magnetic field.

Next, the evolution of magnetic islands is investigated. First, in accordance with Figure 2, islands are categorized into four quadrants, Q1–4, as shown in Figure 6b. The upper right corner, of the plot ( ∆A>0and∆ψ>0) contains islands whose relative cross‐sectional area increases with growth of relative magnetic flux content, as suggested by the linear fit shown in dashed black line, ∆A/A [s^−1^] = 2.16 ( $\pm {\bar{\sigma }}_{S}$) ∆ψ/ψ [s^−1^] – 0.03 $\left(\pm ,{\bar{\sigma }}_{I}\right)$, where $\pm {\bar{\sigma }}_{S}$ = 6.4 × 10^−2^ s and $\pm {\bar{\sigma }}_{I}$ = 1.9× 10^−3^ s^−1^ are the standard errors ( $\bar{\sigma }$ = σ/ $\sqrt{n}$, where σ and *n*, respectively, represent the standard deviation and bin population size (Akhavan‐Tafti, Slavin, Eastwood, et al., 2019) for the derived slope and intercept values, respectively. The linear fits were found using an orthogonal distance regression method using visible data points only. Here the magnetic flux content and cross‐sectional area are normalized to account for variations in island physical properties associated with island size (Akhavan‐Tafti et al., 2018, Akhavan‐Tafti, Slavin, Eastwood, et al., 2019; Hoilijoki et al., 2019).

In Figure 6c, the relative change in the magnetic islands' magnetic flux is investigated as a function of normalized reconnection rate, ℛ. The normalized reconnection rate is determined at the dominant X‐point, that is, lowest magnetic flux function. We find that the majority of magnetic islands experience normalized reconnection rates between 0.05 and 0.15 and that the average reconnection rate is about 0.07. We also find that most coalescence events, shown in red circles, are located within this region. At lower reconnection rates, ℛ < 0.05, magnetic flux is reduced due to reconnection, that is, “erosion.” In contrast, the islands' normalized magnetic flux content, ∆ψ/*∆t*  > 0, is enhanced at higher reconnection rates, ℛ > 0.08. The linear fit, shown in black dashed line, further indicates that as expected, the island's magnetic flux content increases with increasing reconnection rate, ∆ψ/*∆t* [Wb/km‐s] = $8.6\;\left(\pm {\bar{\sigma }}_{S}\right)\;ℛ+0.5\;\left(\pm {\bar{\sigma }}_{I}\right)$, where ${\bar{\sigma }}_{S}$ = 3.4× 10^−1^ and ${\bar{\sigma }}_{I}$ = 3.9× 10^−2^ Wb/km‐s.

Based on the above statistics, the cross‐sectional areas of subsolar islands evolve, on average, via reconnection as follows:

(1) ∆A∆tkm2s≅Akm2ψWb−12.28.6ℛ+0.5±σ¯×103

where *A*, ψ, and ℛ denote, respectively, the FTE cross sectional area (in units of km^2^) and magnetic flux content and the normalized reconnection rate at an adjacent X‐line, assuming steady and continuous reconnection X‐line. The propagated standard error $\bar{\sigma }$ = 1.5 Wb/s.

Equation 1 provides likely an *upper threshold* for the rate of change of the cross‐sectional area of subsolar magnetopause FTEs. The 2‐D nature of the simulation grid requires all interplanetary magnetic flux to reconnect with the magnetosphere at the magnetopause, likely resulting in overestimation of the reconnected flux and, therefore, faster FTE growth.

The purely southward and fast upstream IMF condition may impact the above statistics and average normalized reconnection rates. Hoilijoki et al. (2019) compared Vlasiator island evolution under southward IMF conditions with and without a *B*_*x*_ component and found that magnetopause reconnection dynamics and island frequency and size change across the northern and southern hemispheres under different upstream IMF conditions. Therefore, future analyses shall investigate island statistics under various upstream IMF conditions to further compare with spacecraft observations (Wang et al., 2006).

### 3.2 MMS Case Study

#### 3.2.1 Fields and Plasma Moments

On 14 December 2015 at 0058–0100 UT, the MMS spacecraft were located dawnward of the subsolar magnetopause, at [9.8, −4.3, −0.8] *R*_*E*, GSE_ on an outbound trajectory. The IMF remains steadily southward throughout the interval with a substantial *y* component (clock angle ~45°).

**Figure 7:**
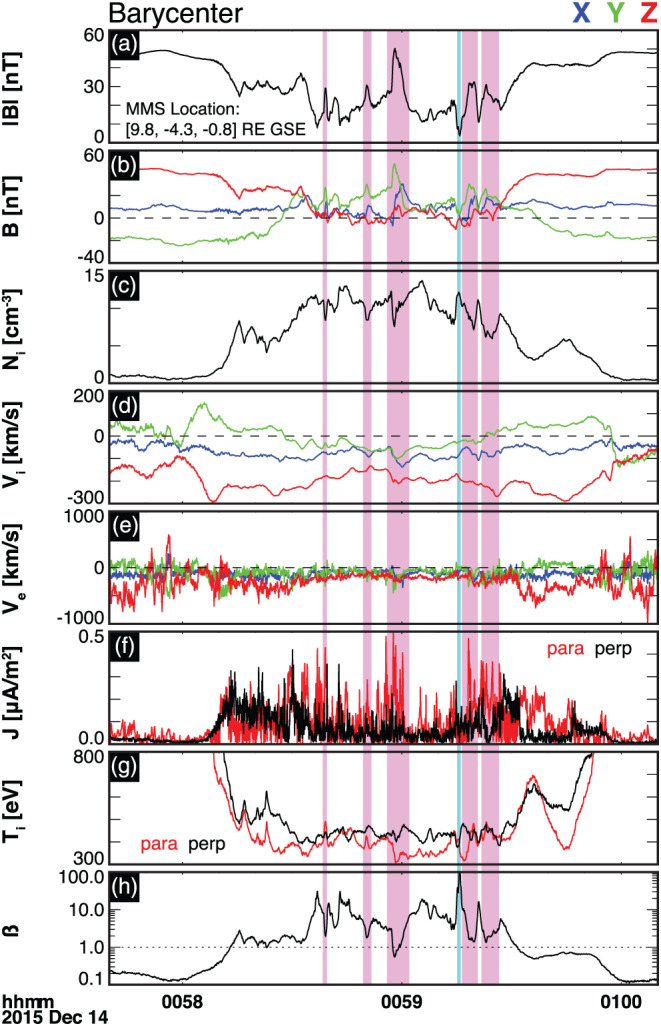
Magnetic field and plasma moments as observed at the barycenter of the four MMS spacecraft for a magnetopause crossing of 14 December 2015—00:57:40–01:00:10 UT. The panels include (a) total magnetic field, (b) magnetic field components in the geocentric solar ecliptic (GSE) coordinates, (c) ion plasma density, (d) ion velocity components, (e) electron velocity components, (f) parallel (red solid line) and perpendicular (black solid line) current density components, (g) parallel (red solid line) and perpendicular (black solid line) ion temperature components, and (h) plasma beta *β*, defined as the ratio of plasma thermal pressure to magnetic pressure. The magenta‐shaded bars indicate the locations of FTEs. The cyan bar indicates the location of a possible reconnection inflow crossing.

In situ magnetic and plasma measurements are shown in Figure 7. From top to bottom, the panels include the following parameters (at the barycenter in the GSE coordinates): (a) total magnetic field, (b) magnetic field components, (c) ion plasma density, (d) ion velocity components, (e) electron velocity components, (f) parallel (red solid line) and perpendicular (black solid line) current density, (g) parallel (red solid line) and perpendicular (black solid line) ion temperature, and (h) plasma beta, *β*, defined as the ratio of plasma thermal pressure to magnetic pressure. This magnetopause crossing is described in detail by Hwang et al. (2018).

At least five localized peaks in the total magnetic field are observed, as shown in magenta bars. The peaks correspond to enhancements in *B*_*y*_ and a bipolar signature in the tangential component of the magnetic field (not shown here). These signatures, together with, plasma density dips (panel c), parallel current density enhancements (panel f), and localized plasma beta dips (panel h) suggest the presence of *d*_*i*_‐scale FTE‐type flux ropes (Akhavan‐Tafti et al., 2018; Akhavan‐Tafti, Slavin, Eastwood*,* et al., 2019; Eastwood et al., 2016). Hwang et al. (2018) stated that the observed FTEs have diameters ranging between, 2.5 < *λ* [*d*_*i*_] < 6.8, where *λ* and *d*_*i*_ (= 75 km) denote the FTE diameter and the local ion inertial length, respectively.

**Figure 8:**
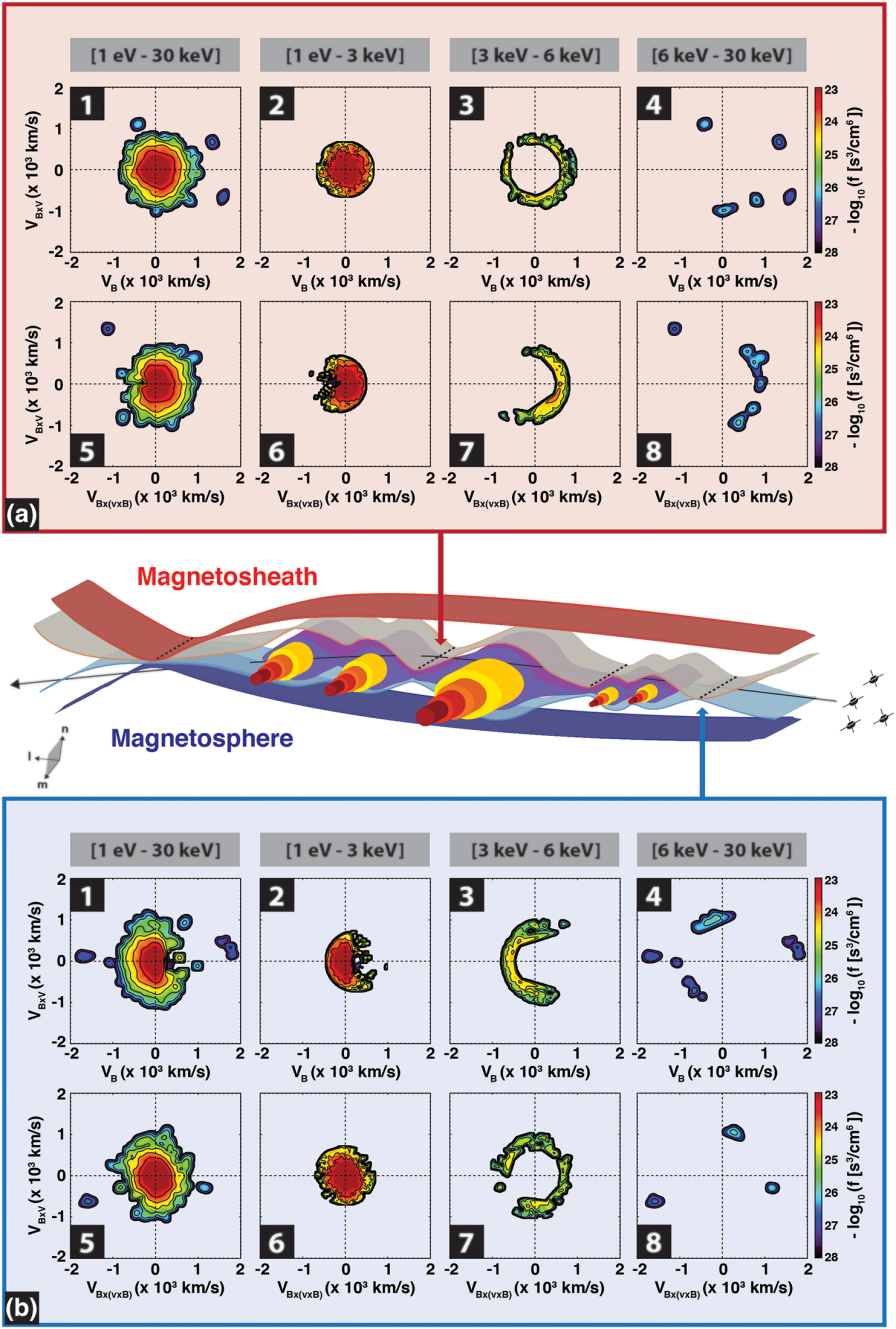
Schematic of the approximate locations and orientations of the observed ion‐scale FTEs, shown as out‐of‐plane cylinders wherein the magnetic field intensity enhances (darker shade) near the FTE core regions, and the adjacent reconnection X‐lines, in the FTE's frame of reference and in the LMN coordinates. The X‐lines are marked by dashed lines. The magnetosheath and magnetospheric magnetic flux are distinguished and shown as red‐ and blue‐shaded surfaces, respectively. The MMS spacecraft trajectory, shown as a black arrow traversing across the structures, is estimated based on the MMS observations. The blue (B) and red (A) arrows/panels represent ion velocity distribution slices in the vicinity of the two observed X‐lines at 00:58:26 and 00:59:15 UT, respectively. The energy bins are divided into four energy bins organized in two rows: *panels* (1–4) the **V**_B_ **− V**_B × V_ slice and panels (5–8) the **V**_B × V_ − **V**_B × (B × V)_ slice, where **V**_B_ represents the velocity along the magnetic field orientation. **V**_B × V_ and **V**_B × (B × V)_ are along (**B** × **V**) and **B** × (**B** × **V**) directions, where **V** is the ion bulk velocity. The energy bins include (panels 1 and 5) 1 eV–30 keV, (panels 2 and 6) 1 eV–3 keV, panels (3 and 7) 3–6 keV, and panels (4 and 8) 6–30 keV. The ion bulk velocity is subtracted from the velocity distribution functions.

The localized dips in magnetic field total coincide with ion flow enhancements and localized field‐aligned current density peaks, suggesting possible reconnection in and outflow region encounters. Hwang et al. (2018) investigated the possible ion jets using the Walén relations (e.g., Sonnerup et al., 1981) and concluded that the MMS spacecraft may have traversed near multiple active X‐lines. They then proposed a magnetic field topology involving multiple ion‐scale FTEs sandwiched between two active X‐lines. Figure 8 provides a schematic illustration of the proposed field geometry. Here the MMS spacecraft are shown to traverse across a reconnecting magnetopause current sheet within which multiple FTEs are detected.

#### 3.2.2 Ion Velocity Distribution Functions

Figure 8 illustrates the approximate locations and geometry of the observed ion‐scale FTEs and the possible X‐lines. The orientation of the FTEs (and the orientation of X‐lines) is along the intermediate eigen vector (M) derived from applying the minimum variance analysis (MVA) on the magnetopause crossing. The eigen vectors (in the Geocentric solar magnetospheric (GSM) coordinates) include: **N** = [0.84, −0.36, −0.41], **M** = [−0.46, −0.87, −0.17], and **L** = [0.30, −0.33, 0.89]. We further investigate the MMS observations of ion VDFs in the vicinity of two independent X‐lines. The VDFs are particle distribution slices in the parallel and perpendicular planes.

In Figure 8, the ion VDFs at the X‐lines are displayed in the ion bulk velocity frame of reference. To achieve this, the components of the bulk ion velocity, **v**_i_, are subtracted from velocity distribution components. Panels 1–4 show VDF slices in the **V**_B_ − **V**_B × V_ plane and panels 5–8 represent the **V**_B × V_ − **V**_B × (B × V)_ plane, where **V**_B_ represents the velocity along the magnetic field orientation, and **V**_B × V_ and **V**_B × (B × V)_, respectively, along the (**B** × **V**) and **B** × (**B** × **V**) directions, where **V** is the ion bulk velocity. The VDF slices are also organized on the basis of ion energy range. From left to right, the panels represent ion measurements: (panels 1 and 5) all energy bins (1 eV–30 keV), (2 and 6) low‐energy ions (1 eV–3 keV), (3 and 7) midenergy ions (3–6 keV), and (4 and 8) high‐energy ions (6–30 keV).

In the vicinity of a first possible X‐line encounter, panel b at the bottom of the figure, marked by a blue arrow, in the **V**_B_ − **V**_B × V_ slice for low‐energy ions (1 eV–3 keV), there exists a nonisotropic population of ions moving antiparallel to B. The low‐energy ion population, 1 eV < *E*_*i*_ < 3 keV, is gyrotropic in the **V**_B × V_ and **V**_B × (B × V)_ slice. However, there exists a nongyrotropic midenergy ion population, 3 < *E*_*i*_ [keV] < 6, with a clear shift in negative **V**_B_ direction in the **V**_B_ − **V**_B × V_ plane, that is, parallel crescent‐shaped population (e.g., Hesse et al., 2014; Wang, Chen, et al., 2016). The high‐energy ion population, 6 < *E*_*i*_ [keV] < 30, appears sparse and likely inconclusive. The counting statistics for the 1 eV < *E*_*i*_ < 6 keV ions further verified (not shown here) that the observed nonisotropic signatures are statistically significant (>4 counts per energy‐angle bin). The 6 < E_i_ [keV] < 30 ion measurements suffer from low‐counting statistics and are, thus, inconclusive.

At a second possible X‐line, marked by a red arrow in panel a at the top of the figure, the low‐energy ions population is found to be symmetric in the **V**_B_ − **V**_B × V_ plane but nongyrotropic, in the **V**_B × V_ − **V**_B × (B × V)_ plane. The mid‐energy ions, 3 < E_i_ [keV] < 6, show an isotropic distribution in the **V**_B_ − **V**_B × V_ plane. However, in the **V**_B × V_ − **V**_B × (B × V)_ plane, the ions form a nongyrotropic, *perpendicular* crescent‐shaped distribution. The high‐energy ion population, 6 < *E*_*i*_ [keV] < 30, remains sparse and likely inconclusive. The counting statistics for the 1 eV < *E*_*i*_ < 6 keV ions further verified (not shown here) that the observed nonisotropic signatures are statistically significant (>4 counts per energy‐angle bin). The 6 < *E*_*i*_ [keV] < 30 ion measurements suffer from low‐counting statistics and are, thus, inconclusive.

The MMS observations further confirm the presence of ion crescent‐shaped distributions in the magnetospheric side of the X‐point, as shown by the Vlasiator simulations. In particular, the perpendicular crescent‐shaped distribution of ions on the magnetospheric side of a Vlasiator X‐point, as shown in panels a4, a5, and b5 in Figure 4, correspond to the MMS‐observed parallel crescent‐shaped midenergy ion distribution, as shown in Figure 8 in panels B3 and B7. The different orientation between the simulated and observed crescent‐shaped distributions may be a result of 2‐D ordinary‐space simulation grid. In particular, the ratio of the out‐of‐plane to in‐plane components of the simulated convection electric field, E ~ (B × V), at the magnetopause may influence the orientation of the crescent‐shaped distributions. This will be further examined in future Vlasiator simulations with 3‐D ordinary‐ and velocity‐space grids.

Similarly, MMS measurements on the magnetosheath side of a possible X‐line encounter further suggest the presence of a perpendicular crescent‐shaped distribution of midenergy ions, panels A3 and A7 in Figure 8. The Vlasiator simulations also show the formation of a nongyrotropic subpopulation of magnetosheath ions in the **V**_B × V_ − **V**_B × (B × V)_ plane, as indicated by a red arrow in Figure 4 in panel b6. These finding also agrees with PIC simulations of asymmetric reconnection (Hesse et al., 2014) and MMS observations (e.g., Wang, Chen, et al., 2006). Previously, global 3‐D MHD with embedded PIC model simulation (MHD‐EPIC) of the Earth's dayside reconnection by Chen, Tóth, et al. (2017) showed similar results.

## 4 Discussion

The main objective of this study is to provide a global perspective on reconnection‐driven mechanisms through which FTEs evolve by utilizing the global Vlasiator simulations and in situ MMS observations. It is, however, important to keep in mind the two major differences between MMS observations and Vlasiator simulations:

1. The upstream solar wind conditions and the spacecraft location at the time of the encounter are somewhat different between in situ observations and the Vlasiator simulations results. In particular, our simulated upstream solar wind conditions lack the IMF *B*_*y*_ (out‐of‐plane) and IMF *B*_*x*_ (Hoilijoki et al., 2019) components. The former component is an essential contributor to the formation of 3‐D flux ropes' core field (e.g., Daughton et al., 2011).
2. The two‐dimensional simulations generate magnetic islands whose central region are characterized by high density (Markidis et al., 2013) and low magnetic field magnitude, contrary to the observed profiles inside flux ropes (Akhavan‐Tafti, Slavin, Eastwood*,* et al., 2019). In fact, magnetic islands collect much of the outflow plasma from adjacent reconnection X‐points. In three dimensions, the outflowing plasma in an X‐line's exhaust region can flow along the out‐of‐plane component of the magnetic field at the outer layers of flux ropes (Chen, Tóth, et al., 2017; Ma et al., 1994; Zhang et al., 2010). Future Vlasiator simulations will further investigate the three‐dimensional aspects of FTEs at the magnetopause.

### 4.1 Reconnection Signatures

The first step in studying reconnection‐driven processes at the magnetopause was to confirm that reconnection signatures were captured by the hybrid‐Vlasov Vlasiator simulations. We find that despite the Vlasiator simulations not fully resolving the ion inertial length in the magnetosheath, the Vlasiator 2‐D simulations and the MMS observations agree in some important and critical aspects, including

1. *FTE generation via multiple X‐line reconnection*. The cyan Vlasiator magnetic island depicted in Figure 3a is generated between two adjacent X‐points. The formation mechanism for the two X‐points is sequential in nature, similar to the SMXR model proposed by Raeder (2006) under southward IMF conditions. The SMXR model suggests that an initial ‐line reconnects field lines. A second X‐line is then formed in the vicinity of the first X‐line, resulting in the formation of an FTE.
  Spacecraft observations (e.g., Trattner et al., 2012) have provided evidence for the multiple X‐line reconnection model by Lee and Fu (1985). For instance, Fear et al. (2008) used Cluster observations to show that the azimuthal extension of an observed FTE could not be explained by the elbow‐shaped FTE model. The Time History of Events and Macroscale Interactions during Substorms (THEMIS) observations have similarly found bidirectional electron flows inside FTEs further suggesting the need for a second X‐line to close the field lines (Hasegawa et al., 2010). Recently, MMS observations provided further evidence for multiple X‐line reconnection at the magnetopause. In particular, Fuselier et al. (2018) showed the presence of adjacent X‐lines at the magnetopause under southward IMF conditions, that is, large IMF clock angles, similar to our simulations' upstream conditions.
  Multiple localized total magnetic field extrema are observed in the in situ magnetic field measurements, as indicated in Figure 7. Further investigation of plasma measurements suggests that |B| peaks are ion‐scale FTEs dispersed within a thin reconnection current sheet, as indicated by the ion jet reversals satisfying the Walén relations (see Hwang et al., 2018) and current density enhancements. The FTEs are likely generated via multiple X‐line reconnection and the associated tearing‐mode instability (e.g., Akhavan‐Tafti et al., 2018; Fuselier et al., 2018; Hwang et al., 2018; Lee & Fu, 1985). In the proposed topology, in agreement with the SMXR model and the Vlasiator simulation results, a second X‐line causes the generation of FTEs.
  Vlasiator simulations further indicate that the reconnection rate can vary between adjacent X‐points. As shown in Figure 5, the reconnection rate at X2 in Figure 3a, located in the exhaust region of X1, increases with time while the reconnection rate at the Southern X‐point is reduced. This may be due to the reconnection exhaust geometry. X2 is sandwiched between two magnetic islands of different scales, while X1 is surrounded by one island and a semiinfinite current sheet. This finding may further underpin the significant role of flux ropes in reconnection dynamics by unblocking the exhaust region and providing a path along which the outflowing plasma can efficiently propagate.
2. *Larmor electric field*. The Larmor electric field is proposed to exist on the magnetospheric side of a collisionless asymmetric reconnection site and should be pointing toward Earth away from the X‐line, *E*_*x*_ < 0 (Malakit et al., 2013). The Vlasiator simulation results indicate the presence of an Earthward electric field extremum, Larmor peak *E*_*x*_ ~ 0.5 mV/m located upstream and on the magnetospheric side of X1, in Figure 3b and 3c. This value is comparable to the theoretically estimated magnitude: *E*_*x*_ ~ 2 mV/m. The global 3‐D MHD‐EPIC simulation of the Earth's dayside reconnection by Chen, Tóth, et al. (2017) showed similar localized Earthward pointing electric fields that precede a strong sunward electric field.
3. *Anisotropic ion distributions in the vicinity of a reconnection site*. The Vlasiator simulations indicate the formation of a perpendicular crescent‐shaped distribution of ions in the magnetospheric inflow region of the X‐points, sometimes associated with the Larmor electric field, such as in Figure 4 panel a4 (e.g., Lapenta et al., 2017). The crescent‐shaped ion population becomes clearer closer to the X‐point, as shown in Figure 4 panel a5. Nongyrotropic, crescent‐shaped ions are also observed during possible reconnection inflow region encounters, as shown in panels a7 and b3 in Figure 8. Further investigation is needed to examine and compare the observed and simulated nonisotropic ion distributions in the magnetosheath as well as the magnetospheric reconnection inflow regions. Furthermore, the roles of upstream solar wind conditions, local energization mechanisms (Akhavan‐Tafti et al., 2019), and the magnetopause convection electric field orientation in determining the properties of anisotropic ion distributions in reconnection inflow regions shall be investigated.

Future investigations will use 3‐D spatial and velocity simulations under more representative upstream conditions to improve the above results.

### 4.2 Magnetic Island Evolution

After confirming that the Vlasiator simulations captured key reconnection signatures, the next step was to determine the relative roles of the different reconnection‐driven mechanisms through which magnetic islands evolve, as described by the introductory Figure 2. A total of 4,786 subsolar islands (|*θ*| < 30°) are identified across time frames. The islands are divided into four main categories depending on their structure and evolution: (1) “two X‐points,” (2) “>2 X‐points,” (3) “Coalescence,” and (4) “Division.” It is concluded that

1. On average, continuous reconnection at adjacent dominant X‐points supplies additional magnetic flux, *∆ψ* > 0, to the outer layers of magnetic islands. The additional supply of magnetic flux further causes an increase (*∆A* > 0) in the cross‐sectional areas of the majority of these islands, as proposed by Akhavan‐Tafti, Slavin, Eastwood, et al., 2019; cf. Figure 1).
2. In 16% of the islands belonging to Category 2, magnetic flux content increases while the islands' cross‐sectional area is reduced, Q4. This suggests that the internal X‐points may contribute to dividing the dominant island into at least two smaller islands, that is, island division. Another possible scenario, as postulated by Akhavan‐Tafti, Slavin, Eastwood, et al. (2019), may include the compression of the island due to the radially inward force from the continuous supply of magnetic flux at adjacent X‐points (*∆A* < 0 and *∆ψ* > 0), before the island grows in dimensions.
3. In the Coalescence category, 42% of the magnetic islands grow in dimensions (Q1 and Q2) while 42% are eroded, that is, reduction in cross‐sectional area (Q1 and Q3). These suggest that as discussed by Fermo et al. (2011), the coalescence process involves the merging of two neighboring islands wherein the smaller of two islands is consumed, that is, eroded (*∆ψ* < 0), by the larger island.
4. The likelihood of identifying island division events is nearly twice that of island coalescence events suggesting that the division of magnetic islands is more common than coalescence (e.g., Øieroset et al., 2011, 2019), at least in two dimensions. While identifying the two processes in spacecraft observations is challenging, one approach in distinguishing flux rope coalescence from division is to investigate the relative structure velocities. In the coalescence process, the two neighboring flux ropes approach each other (in the flux ropes' frame of reference), whereas in the division process the structure velocity vectors point in opposite directions (moving away from one another).
5. The proposed classification in Figure 2 oversimplifies the island dynamics by neglecting the fact that one or two of the processes could occur concurrently. For instance, in Category 3, the two X‐points on the outer edges of the two coalescing islands can and do continue to supply additional magnetic flux and plasma to the outer layers of the two islands.
6. Continuous reconnection, Category 1, is the dominant contributor to island evolution (e.g., Akhavan‐Tafti, Slavin, Eastwood*,* et al., 2019; Paschmann et al., 1982). However, this result may also be due to the 2‐D nature of our simulations and/or our island selection criteria wherein islands are defined as “O points” sandwiched between two “X‐points.”

Future 3‐D spatial and velocity simulations will reexamine the relative roles of the reconnection‐driven FTE evolution mechanisms.

### 4.3 FTE Growth Rate

Finally, the Vlasiator simulations' global perspective on the evolution of magnetic islands via reconnection‐driven mechanisms can be further utilized to inform in situ observations. In particular, spacecraft observations are not capable of studying the temporal evolution of individual FTEs. Therefore, global simulations can further be used to interpret spacecraft observations. In this section, the normalized reconnection rate as measured by the MMS spacecraft in the vicinity of a possible reconnection inflow region located between two ion‐scale FTEs is used in combination with the Vlasiator island statistics in order to estimate the rate at which the two FTEs may grow via reconnection.

The Vlasiator simulations indicate that the change in magnetic island magnetic flux content, *∆ψ*, due to continuous reconnection at adjacent X‐lines is a function of normalized reconnection rate. In 2‐D, the magnetic flux content is described as the enclosed in‐plane flux within an area and, therefore, it is, in practice, different from in situ observations. However, the rate of change in magnetic flux, *∆ψ* [Wb/s‐km], is, to a first‐order approximation, similar in 2‐D simulation results and spacecraft observations. The rate of change in magnetic flux is determined in the reconnection plane (per X‐line length) along the X‐line.

Hoilijoki et al. (2019) confirmed that the size distribution of Vlasiator magnetic islands at the magnetopause is similar to that of the subsolar FTEs as observed by MMS and reported by Akhavan‐Tafti et al. (2018). Here we determine the reconnection rate ℛ of a possible X‐line positioned near an observed *d*_*i*_‐scale FTE to estimate the FTE's rate of growth based on the Vlasiator simulation island statistics (Equation 1 in section 3.1.2).

**Figure 9:**
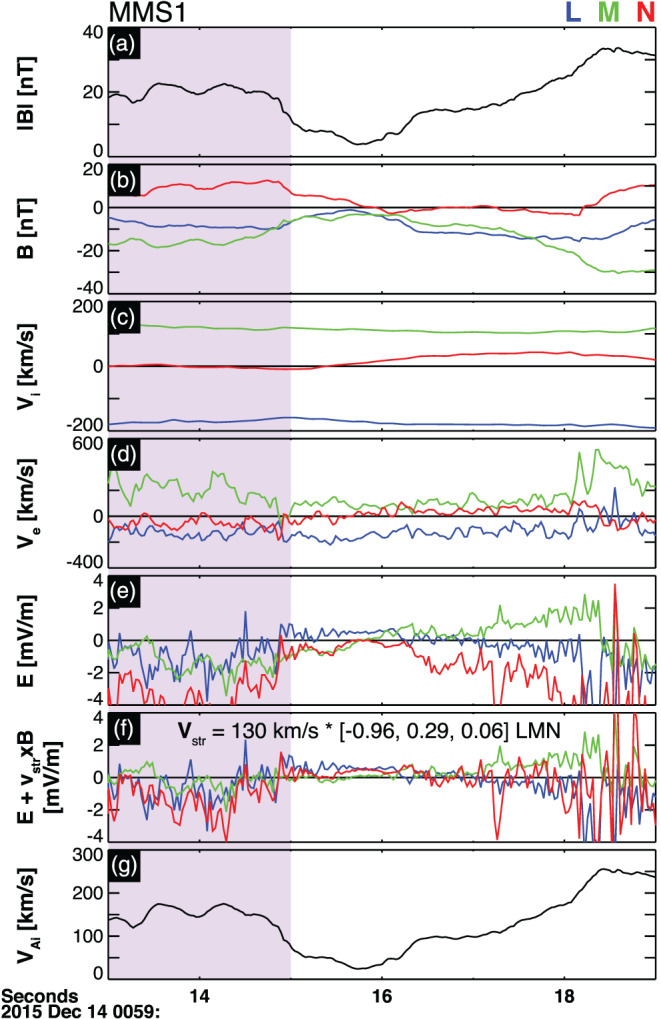
Fields and plasma moments in the vicinity of a possible reconnection inflow crossing in LMN coordinates as observed by MMS 1: (a) magnetic field magnitude, |B|, (b) magnetic field components, (c) ion velocity components, (d) electron velocity components, (e) electric field components in the spacecraft's frame of reference, (f) corrected electric field components in the current sheet's frame of reference, and (g) Alfvén velocity. The normalized reconnection rate is estimated from the fields and plasma signatures in the purple‐shaded region.

Figure 9 shows the observed fluxgate magnetometer magnetic field vectors, the bulk FPI ion and electron velocities, and the electric dual probe electric field vectors at the location of MMS1 in the vicinity of a possible X‐line encounter sandwiched between two of the observed FTEs, marked with a cyan bar in Figure 7. The vectors are transformed into the LMN coordinate system where the LMN eigen vectors (in the GSM coordinates) are as follows: **N** = [0.84, −0.36, −0.41], **M** = [−0.46, −0.87, −0.17], and **L** = [0.30, −0.33, 0.89] (Hwang et al., 2018). High‐resolution electric field measurements are boxcar averaged to match the cadence of the FPI electron measurements, that is, 30 ms. Using MVA and the timing analysis techniques, the X‐line is found to convect at a normal velocity ***v***_*structure*_ [km/s] = 130 × [−0.96, 0.29, 0.06] LMN, as reported by Hwang et al., (2018). Therefore, the electric field vector in the X‐line's frame of reference can be estimated as **E**_*X‐line*_ = **E** + **v**_*X‐line*_ × **B** (panel f).

The observed MMS estimated out‐of‐plane component of electric field, *E*_*M*_, averaged at the vicinity of the X‐line within the purple‐shaded interval is *E*_*M*, *obsv*_ = 0.2 mV/m. This corresponds to an averaged normalized reconnection rate, ℛ = *E*_*M*_/*V*_*Ai*_ *B*_*MSH*_ = 0.18, where *V*_*Ai*_ and *B*_*MSH*_ are the average upstream Alfvén velocity (panel g) and magnetic field magnitude (panel a), respectively (e.g., Genestreti et al., 2018; Liu et al., 2017). Based on the Vlasiator simulation fit of *∆ψ*/*∆t* [Wb/km‐s] = 8.6ℛ+0.5, where ℛ represents the rate of reconnection, it is concluded that, to a first‐order approximation, the observed *d*_*i*_‐scale FTE (*ψ* = 4.4 kWb) can gain magnetic flux at rate <+9.0 kWb/s‐km due to continuous supply of magnetic flux at the adjacent X‐lines. At this rate, this FTE will contain 1 MWb of magnetic flux (e.g., Wang et al., 2005) in nearly 2 min.

The global Vlasiator simulations results are further utilized to determine the average rate at which FTEs grow (*∆A*/*∆* *t*) at the magnetopause. From the MMS‐observed normalized rate of reconnection rate, ℛ = 0.18, and ion‐scale FTE sizes and magnetic flux contents, it is determined that the ion‐scale FTE observed in the vicinity of an X‐line may grow in size at rate:

∆A∆tλ=6.8diℛ=0.18ψ=4.4kWb≤+2.1±0.7×105km2s=+3.1±0.1×10−1RE2min.

At this growth rate, the ion‐scale FTE will grow Earth sized, r= 1 *R*_*E*_ (e.g., Eastwood et al., 2012), in $10_{-2}^{+5}$ min due to steady and continuous reconnection at adjacent X‐lines while convecting away from the subsolar region along the magnetopause. This estimated growth duration is comparable to the transport time for an FTE forming at the subsolar magnetopause to reach the high‐latitude magnetopause, that is, ~10 min (e.g., Owen et al., 2001). Future studies will focus on the evolutions of ion‐scale (e.g., Sun et al., 2019) and large‐scale flux ropes (e.g., Slavin et al., 1995, 2003) in the Earth's magnetotail.

As noted previously, the aboveestimated FTE growth rate is likely an *upper threshold* for the rate of change of the cross‐sectional area of subsolar magnetopause FTEs. The 2‐D nature of the simulation grid requires all interplanetary magnetic flux to reconnect with the magnetosphere at the magnetopause, probably resulting in overestimation of the reconnected flux and, therefore, faster FTE growth. Additionally, accurate determination of the reconnection plane orientation is key for measuring reconnection rate (e.g., Genestreti et al., 2018). In this study, the MVA technique is used to determine the reconnection plane. As provided in the supporting information Table S1, we rely on the fact that the applications of MVA on magnetic field (MVA B) and electron velocity (MVA Ve) measurements for the this partial magnetopause crossing generate similar intermediate ( $\hat{M}$) eigen vectors. However, there can still remain large uncertainty in determining the accurate reconnection plane which can impact the reconnection rates, and therefore, the FTE growth rate provided in this study.

It is concluded that the difference between the average FTE size observed at the subsolar magnetopause by MMS (Akhavan‐Tafti et al., 2018) and at high‐latitude and flank regions by Cluster (e.g., Fermo et al., 2011; Wang et al., 2005) is most likely due to their orbits and different FTE sampling populations. As suggested by Akhavan‐Tafti et al. (2018), FTEs grow while convecting away from the subsolar region where they are most likely formed. Therefore, MMS, due to its near‐equatorial orbit, is expected to observe smaller‐scale FTEs soon after formation, hence, smaller average FTE size compared to Cluster, due to its polar orbit. Further investigation of the Cluster magnetic field data is required to confirm this conclusion, since the study by Wang et al. (2005) did not account for small‐scale FTEs (4 s cadence magnetometer data).

Characteristic scale lengths, including the ion inertial length, determine the microscale reconnection physics. However, simulating global simulation systems with a broad range of temporal and spatial dynamical scales remains quite challenging (e.g., Tóth et al., 2017). The magnetosheath ion inertial length (*d*_*i*_ ~ 150 km at the magnetopause under the stated upstream conditions) is not resolved in the Vlasiator's simulation grid (spatial grid resolution = 300 km) which may impact the microphysics of reconnection, and therefore, the reconnection‐driven dynamics and rates provided in this study. Nevertheless, Vlasiator is shown to capture reconnection ion kinetics, despite not resolving the relevant spatial scales. Future investigations shall revisit Vlasiator reconnection in simulations with improved spatial resolutions.

## 5 Conclusion

The hybrid‐Vlasov Vlasiator simulations of multiple X‐point reconnection at the subsolar region are compared with MMS observations of two neighboring X‐lines within which at least five ion‐scale FTEs are identified. The signatures of multiple X‐line reconnection are found in both the Vlasiator simulations and the MMS observations. Nonisotropic ion distributions are observed in MMS observations in two possible reconnection inflow region encounters. These nonisotropic (crescent shaped) ion distributions are in good agreement with the Vlasiator simulations, indicating that despite not fully resolving the ion inertial length in the magnetosheath, the Vlasiator code captures key reconnection signatures.

We further investigate the evolution of the Vlasiator's two‐dimensional magnetic islands due to magnetic reconnection. Magnetic islands at the low‐latitude magnetopause (polar angle, |*θ*| < 30°) are found to grow mainly due to continuous reconnection at adjacent X‐points. It is also shown that magnetic islands further evolve due to coalescence, erosion, and division. The relationship between the normalized rate of reconnection at an adjacent X‐point and the change in the islands' enclosed magnetic flux is determined.

The average rate at which magnetic flux is added to the outer layers of magnetic islands due to continuous reconnection at adjacent X‐points is determined. Based on our statistical analysis of magnetic islands in the Vlasiator code, magnetic islands grow at <+0.3 *R*_*E*_^2^/min. At this rate, a subsolar MMS‐observed *d*_*i*_‐scale FTE is estimated to grow Earth sized within ~10 min while convecting away from the reconnection sites along the magnetopause. The estimated growth time is comparable to the average transport time for FTEs formed in the subsolar region to reach the high‐latitude magnetopause and flanks. More importantly, the Vlasiator island statistics provide an equation for estimating the rate of FTE growth (*∆A*/*∆* *t*) that is based on physical parameters that can be (directly or indirectly) measured by single or multispacecraft observations, including FTE size (*A*), magnetic flux content (*ψ*), and normalized reconnection rate ( ℛ). Vlasiator reconnection dynamics shall be revisited in future simulations with improved spatial resolutions.

Finally, it is concluded that the discrepancy in the average FTE size at the magnetopause between the Cluster and MMS observations is most likely the result of their different orbits, wherein MMS mainly observes the newly formed FTEs at the subsolar region near the magnetic equator while Cluster detects grown FTEs farther away from the subsolar region at higher‐latitude magnetopause and flanks. Further investigation of the Cluster magnetic field measurements is required to confirm this conclusion.

## Supporting information

Supporting Information S1

Table S1

Movie S1

Movie S2
