# Supplementary material for: Comparative Analysis of the Vlasiator Simulations and MMS Observations of Multiple X‐Line Reconnection and Flux Transfer Events
Source: J Geophys Res Space Phys. 2020 Jul 22;125(7):e2019JA027410. doi: 10.1029/2019JA027410 (PMC7507759; doi:10.1029/2019JA027410)
Supplement: Supplementary file 1 — Supporting Information S1 [file JGRA-125-e2019JA027410-s001.docx]

**Comparative Analysis of the Vlasiator Simulations and MMS Observations of Multiple X-Line Reconnection and Flux Transfer Events**

**M. Akhavan-Tafti**^1,2^, M. Palmroth^3^, J. A. Slavin^1^, M. Battarbee^3^, U. Ganse^3^, M. Grandin^3^, G. Le^4^, D. J. Gershman^4^, J. P. Eastwood^5^, J. E. Stawarz^5^

^1^ Climate and Space Sciences and Engineering, University of Michigan, Ann Arbor, MI, USA.

^2^ Laboratoire de Physique des Plasmas (LPP), CNRS, École Polytechnique, Sorbonne Universié, Universié Paris-Saclay, Observatoirede Paris, PSL Res Universié, Institut Polytechnique de Paris, Palaiseau, France

^3^ Department of Physics, University of Helsinki, Helsinki, Finland.

^4^ NASA Goddard Space Flight Center, Greenbelt, MD, USA.

^5^ Blackett Laboratory, Imperial College, London, UK.

**Content of this file**

1. Video S1
2. Video S2
3. Table T1

**Introduction**

The supplemental videos, Video S1 and S2, demonstrate the temporal evolutions of electric field profiles and ion velocity distribution functions at two Vlasiator’s dayside magnetopause X-points, ‘X1’ and ‘X2’. The supplemental table, Table S1, lists the reconnection plane intermediate eigen vectors from applying the minimum variance analysis technique on various vector measurements.

**Video S1:** Vlasiator electric field profile and ion velocity distribution functions across X1. The panels include: *top left*) Simulation frame of a magnetic island located in the northern hemisphere. The contours represent the XZ-plane magnetic field and the color bar shows the magnitude and the direction of electric field along X_GSM_. The red dashed line denotes a virtual spacecraft outbound trajectory through the X-point. *bottom left*) One-dimensional E_x_ profile along the virtual spacecraft’s trajectory. VDF cuts along the virtual spacecraft’s trajectory in $\text{V}_{\text{B}}\text{ - }\text{V}_{\text{B×V}}$(*top right*) and $\text{V}_{\text{B×V}}\text{ - }\text{V}_{\text{B×(B×V)}}$ (*bottom right*) diagrams, where **V**_B_ represents the velocity along the magnetic field orientation. $\text{V}_{\text{B×V}}$ and $\text{V}_{\text{B×(B×V)}}$ are along ($\text{B }\text{×}\text{ V}$) and $\text{B}\text{ × (}\text{B }\text{×}\text{ V}\text{)}$ directions, where **V** is the ion bulk velocity.

**Video S2:** Vlasiator electric field profile and ion velocity distribution functions across X2. The panels include: *top left*) Simulation frame of a magnetic island located in the northern hemisphere. The contours represent the XZ-plane magnetic field and the color bar shows the magnitude and the direction of electric field along X_GSM_. The red dashed line denotes a virtual spacecraft outbound trajectory through the X-point. *bottom left*) One-dimensional E_x_ profile along the virtual spacecraft’s trajectory. VDF cuts along the virtual spacecraft’s trajectory in $\text{V}_{\text{B}}\text{ - }\text{V}_{\text{B×V}}$(*top right*) and $\text{V}_{\text{B×V}}\text{ - }\text{V}_{\text{B×(B×V)}}$ (*bottom right*) diagrams, where **V**_B_ represents the velocity along the magnetic field orientation. $\text{V}_{\text{B×V}}$ and $\text{V}_{\text{B×(B×V)}}$ are along ($\text{B }\text{×}\text{ V}$) and $\text{B}\text{ × (}\text{B }\text{×}\text{ V}\text{)}$ directions, where **V** is the ion bulk velocity.

**Table S1:** The reconnection plane intermediate eigen vector ($\hat{\text{M}}$) in GSE and spherical coordinates. The intermediate vectors are derived independently using the Minimum Variance Analysis (MVA) technique applied on spacecraft measurements of magnetic field (B), electric field (E), electron velocity (Ve), and ion velocity (Vi) between 201512/14-00:59:13-19 UT.
