# Supplementary material for: Comparative Analysis of the Vlasiator Simulations and MMS Observations of Multiple X‐Line Reconnection and Flux Transfer Events
Source: J Geophys Res Space Phys. 2020 Jul 22;125(7):e2019JA027410. doi: 10.1029/2019JA027410 (PMC7507759; doi:10.1029/2019JA027410)
Supplement: Supplementary file 2 — Table S1 [file JGRA-125-e2019JA027410-s002.docx]

**Table S1:** The reconnection plane intermediate eigen vector ($\hat{\text{M}}$) in GSE and spherical coordinates. The intermediate vectors are derived independently using the Minimum Variance Analysis (MVA) technique applied on spacecraft measurements of magnetic field (B), electric field (E), electron velocity (Ve), and ion velocity (Vi) between 201512/14-00:59:13-19 UT.

|  | **Intermediate (**$\hat{\text{M}}$**) eigen vector**  **(GSE Coords.)** | | | **Intermediate (**$\hat{\text{M}}$**) eigen vector**  **(Polar Coords.)** | |
| --- | --- | --- | --- | --- | --- |
|  | $\hat{\boldsymbol{x}}$ | $\hat{\boldsymbol{y}}$ | $\hat{\boldsymbol{z}}$ | $\boldsymbol{\theta}$ **[deg]** | $\boldsymbol{\phi}$ **[deg]** |
| **MVA B** | 0.90 | -0.43 | 0.10 | 84.26 | 115.47 |
| **MVA E** | 0.62 | -0.60 | 0.52 | 58.67 | 126.87 |
| **MVA Ve** | 0.94 | -0.35 | 0.02 | 91.15 | 110.49 |
| **MVA Vi** | -0.07 | 0.02 | 1.00 | 00.00 | 88.85 |
